# Preventing nasal airway collapse with irradiated homologous costal cartilage versus expanded polytetrafluoroethylene: a novel animal model for nasal airway reconstruction

**DOI:** 10.1038/s41598-019-42947-8

**Published:** 2019-04-30

**Authors:** Cheng-I Yen, Jonathan A. Zelken, Chun-Shin Chang, Hung-Chang Chen, Shih-Yi Yang, Shu-Yin Chang, Jui-Yung Yang, Shiow-Shuh Chuang, Yen-Chang Hsiao

**Affiliations:** 1 Department of Plastic and Reconstructive Surgery, Chang Gung Memorial Hospital, College of Medicine, Chang Gung University, Taipei, Taiwan; 2 Private Practice, Newport Beach, California, USA

**Keywords:** Cartilage, Experimental models of disease

## Abstract

Airway collapse can occur when the forces of inhalation overpower the strength of the nasal lining flap. The authors established an animal model of the reconstructed nasal airway, and examined mechanical properties of tissue composites based on various materials. Twenty-three Sprague-Dawley rats were divided into three experimental groups: control (n = 5), irradiated homologous costal cartilage (IHCC, n = 10), and expanded polytetrafluoroethylene (ePTFE, n = 8). Two dorsal skin flaps represented nasal lining and skin envelope. No framework, an IHCC or ePTFE rim graft was used as framework. At three weeks, changes in the cross-sectional area of the lining flap were measured when negative pressure was applied. En-bloc specimens containing the graft and soft tissue were examined for histological change and tissue ingrowth. Reduction of cross-sectional area with simulated inhalation was 87.74% in the control group, 82.76% (IHCC), and 67.29% (ePTFE). Cross-sectional reduction was significantly less in ePTFE group than control group (p = 0.004) and IHCC group (p = 0.001). The difference was not significant in the control and IHCC groups. There was histologic evidence of tissue ingrowth in the ePTFE group. This novel animal model of nasal airway reconstruction supports the use and potential benefit of using ePTFE for prevention of airway collapse.

## Introduction

The nose is quite difficult to reconstruct with satisfactory results because of its complex topography and robust structural integrity^1^. Full thickness defects of the nose either after skin cancer extirpation, congenital deformity, trauma, infection, or burn injury, require three components to be replaced in reconstruction: skin, framework, and nasal lining. To achieve this, soft tissue must sandwich a framework to create form and a patent airway^1–8^. However, the pliable skin of the forearm, a common donor to replace soft tissue^1–5^, is prone to collapse. With inhalation, negative pressure in the airway can overcome the support of the cartilage framework and tissue, and resulted in the collapse of airway, which may lead to airway obstruction or difficulty of breathing in our clinical observation **(**Video 1). To treat this, we rely on nasal stents to avoid flap collapse and stenosis^1,2,4^. Although cartilage remains the gold standard in nasal reconstruction, alloplast support may confer better strength and airway protection. In this study, irradiated homologous costal cartilage (IHCC) was compared to expanded polytetrafluoroethylene (ePTFE, Gore-Tex) as a framework medium.

Because ePTFE is biocompatible, surrounding tissue is expected to grow into its microporous structure, and to create a bond between the lining flap and ePTFE^9–14^. The authors hypothesize that ePTFE is superior to cartilage for prevention of flap collapse. If the results suggest improved mechanical properties, this would support a paradigm shift in human nasal reconstruction favoring alloplast materials.

## Results

There were seven rats that died or suffered flap demise that were excluded. 23 rats previously divided into experimental and control groups were included. These rats survived during the experiment and there was no partial or total flap necrosis, wound dehiscence, hematoma, cellulitis or graft extrusion.

**Figure 1:**
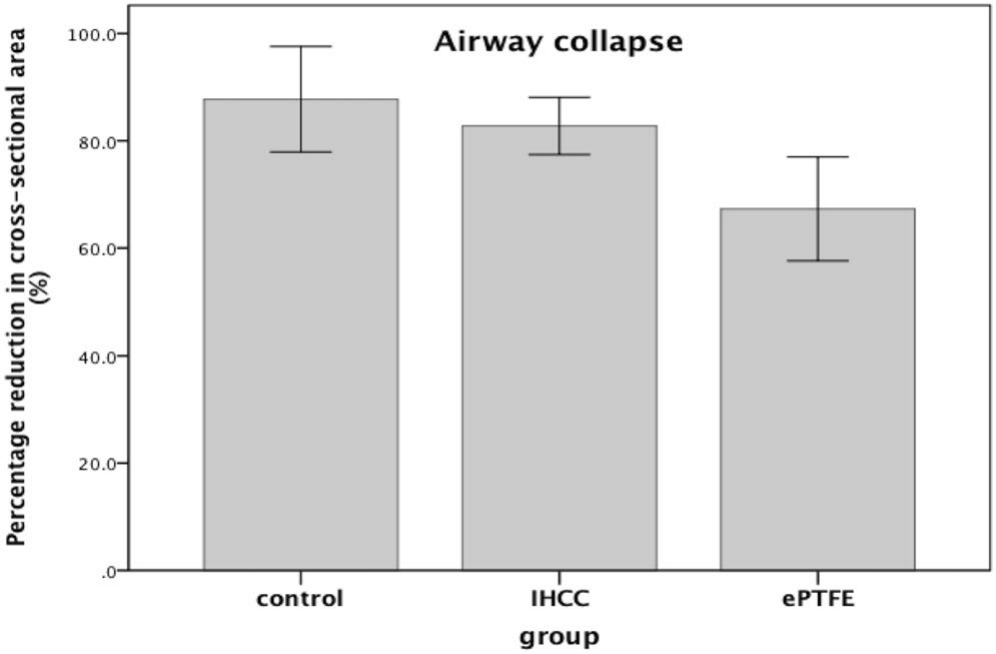
The reduction of cross-sectional area was 87.74% in the control group, 82.76% in the IHCC group, and 67.29% in ePTFE group.

There was a cross-sectional area reduction 87.74 ± 9.83% in control group after suction was applied to the airway. Reduction was 82.76 ± 5.34% in the IHCC group, and 67.29 ± 9.68% in the ePTFE group **(**Fig. 1**)**. The ePTFE group showed significantly less reduction compared to the control group (p = 0.004) and IHCC group (p = 0.001). There was no difference in cross-sectional area reduction after negative pressure between the control and IHCC groups (p = 0.219).

**Figure 2:**
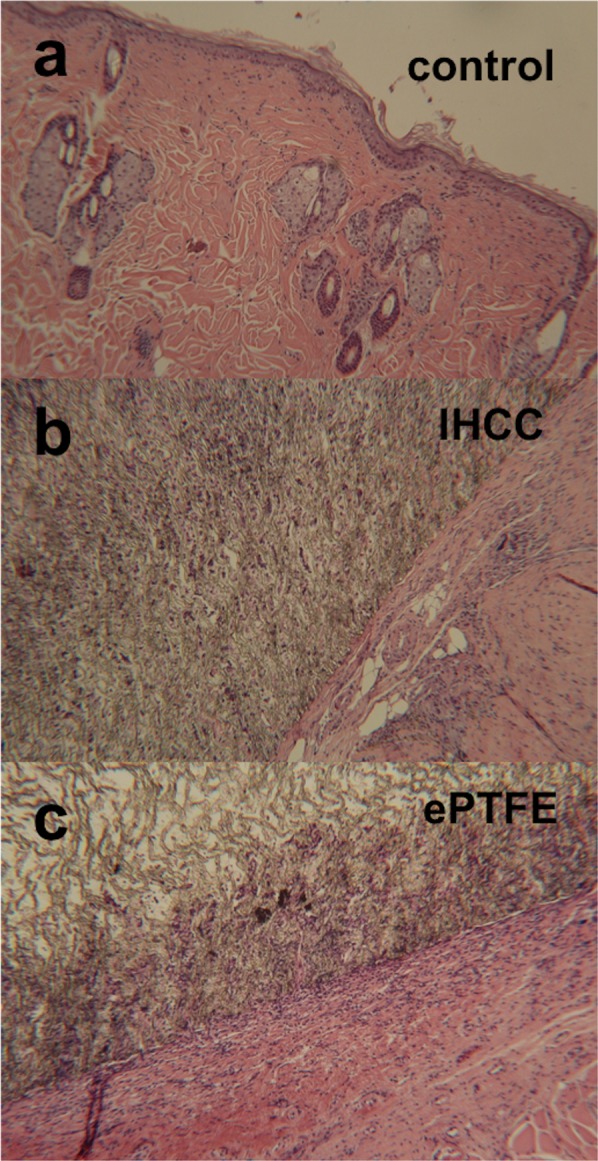
Histological evaluation with hematoxylin and eosin (H&E) stain demonstrated connective tissue ingrowth and abundant infiltration and deposition of fibroblasts and collagen in the ePTFE group. (**a**) control group; (**b**) IHCC group; (**c**) ePTFE group.

Histologic evaluation demonstrated connective tissue ingrowth with abundant infiltration and deposition of fibroblasts and collagen in the ePTFE group. This was neither seen in the control nor the IHCC group **(**Fig. 2**)**.

## Discussion

The most important aspect of nasal reconstruction is effective reproduction of each component: skin, framework, and lining^5^. In complex nasal defect reconstruction, it may be necessary to transfer free tissue when the local tissue is not available or enough^1–8^. The radial forearm flap is favored for its thin and pliable soft tissue, and provides lining without obstructing the nasal airway^1–5^. At Chang Gung Memorial Hospital, the ulnar forearm flap is sometimes preferred for superior donor site cosmesis compared to radial forearm flap^4^. The literature is supportive of both options^1–8^, but there is rare mention of late airway collapse due to inadequate framework support. Furthermore, no animal models or clinical reports objectively address airway obstruction after nasal reconstruction^15–21^. To our knowledge, the animal model demonstrated is the first to simulate a hollow cylinder and the layer components of the wall to mimic the structure of human nasal airway and the dynamic changes that occur during inhalation. We compared different materials for construct stability and prevention of airway collapse.

The framework is crucial for restoring aesthetics and function after nasal reconstruction by fortifying the construct and maximizing airflow^1^. Lateral alar structural grafts are designed to prevent alar retraction and external valve collapse during inspiration. Cartilage is typically harvested from the nasal septum, auricular concha or rib^1,2,4^. Autologous materials are preferred, but are not immune to absorption and warping. Donor site morbidity, increased operative time and insufficient donor material are important considerations^11,22–24^. Cadaveric homograft cartilage may be used and displays similar characteristics as autologous tissue^22–26^, but the rate of absorption is greater than with autogenous tissue^2,23^. Despite a successful framework reconstruction using cartilage, it is possible for the framework to collapse during inhalation. Autologous rib cartilage may not be sufficiently strong to support the airway in the long term. This may be attributable to its histologic character or to inadequate soft tissue ingrowth into the cartilage.

ePTFE, marketed as Gore-Tex (W.L. Gore Associates, Inc., Phoenix, AZ), was developed by Gore in 1969, has a 20 year track record of biocompatibility and favorable results^9–14,27^. The material demonstrates low tissue reactivity, confers stability, does not resorb, and is chemically inert. For these reasons, it is popular in rhinoplasty surgery^9,10^. ePTFE has a microstructure of interconnected nodes and fibrils that create pores with diameters from 10 to 40 μm^9^. Morphologic and histologic changes in surrounding tissues have been reported using a Gore-Tex implant in animal models^9^. When connective tissue ingrowth occurs, deposition of collagen in the intermodal spaces occurs^9–14^. The complications in rhinoplasty using Gore-tex ranged from 1.9% to 10%, compared to silicone implants which ranged from 2 to 7% and autologous costal cartilage which ranged from 0 to 39.5%^9,27–29^.

Histologic results from this study corroborate findings from previous reports^9–14^. Connective tissue was incorporated into ePTFE, which is likely responsible for its bond to surrounding tissues, and for the support and stability of the lining flap. These findings support the idea that ePTFE may be superior to autograft and allograft in preventing flap airway collapse.

The authors used IHCC instead of murine rib cartilage to better simulate autologous human rib cartilage in nasal reconstruction. Autologous rib cartilage is the most common donor for framework reconstruction. There are larger, more evenly distributed, and uniform chrondrocytes, collagen, and proteoglycans in autologous costal cartilage compared to IHCC^23^, but there is preservation of lacunar morphology in IHCC^25^. The authors believe IHCC is the best substitute for autogenous rib cartilage compared to other materials^24–26^. It is readily available and murine rib cartilage is too small to simulate human nasal reconstruction. Not to mention, there is technical difficulty and increased operative time involved with murine rib harvest^30,31^.

Alloplasts such as porous polyethylene (PPE, Medpor®) are widely available for clinical applications. The authors chose ePTFE for this study for its well-known tissue ingrowth characteristics and softer consistency compared to PPE. In future study, the authors intend to create more elaborate airway models in larger animals that allow for autograft rib cartilage to be used, and to study other alloplasts.

### Limitation

We assumed complete viability of the tubed flap in all animals. Subclinical construct breakdown would influence the outcome of the experiment. Although we standardized the dissection plane as harvesting the flap above the muscle layer for all rats, the tissue thickness was hardly to be unified. To optimize the health of the constructs, animals were studied no later than three weeks post-experiment and a protective device was placed to prevent flap destruction. Long term changes, including framework resorption and collagen replacement were not studied.

The negative pressure of 60–70 mmHg is much higher than the 150 Pa (~1 mmHg) transmural pressure that causes nasal valve collapse in human patients with nasal airway obstruction. The authors would have preferred to quantify the threshold pressure for flap collapse between the three groups, but this model did not allow for this because of a uniform vacuum pressure. Besides, drawing the airway perimeter manually and selecting the cross-sectional area manually on the Image J software were also a limitation in quantifying the reduction in lumen cross-sectional area. Moreover, this paper did not account for the different mechanical properties of ePTFE and IHCC. Still, this study supports that ePTFE rim grafts are more resilient than cartilage in a rat model. Based on these findings, additional study in human clinical trials may be warranted.

## Methods

### Animal Model and Study Design

**Figure 3:**
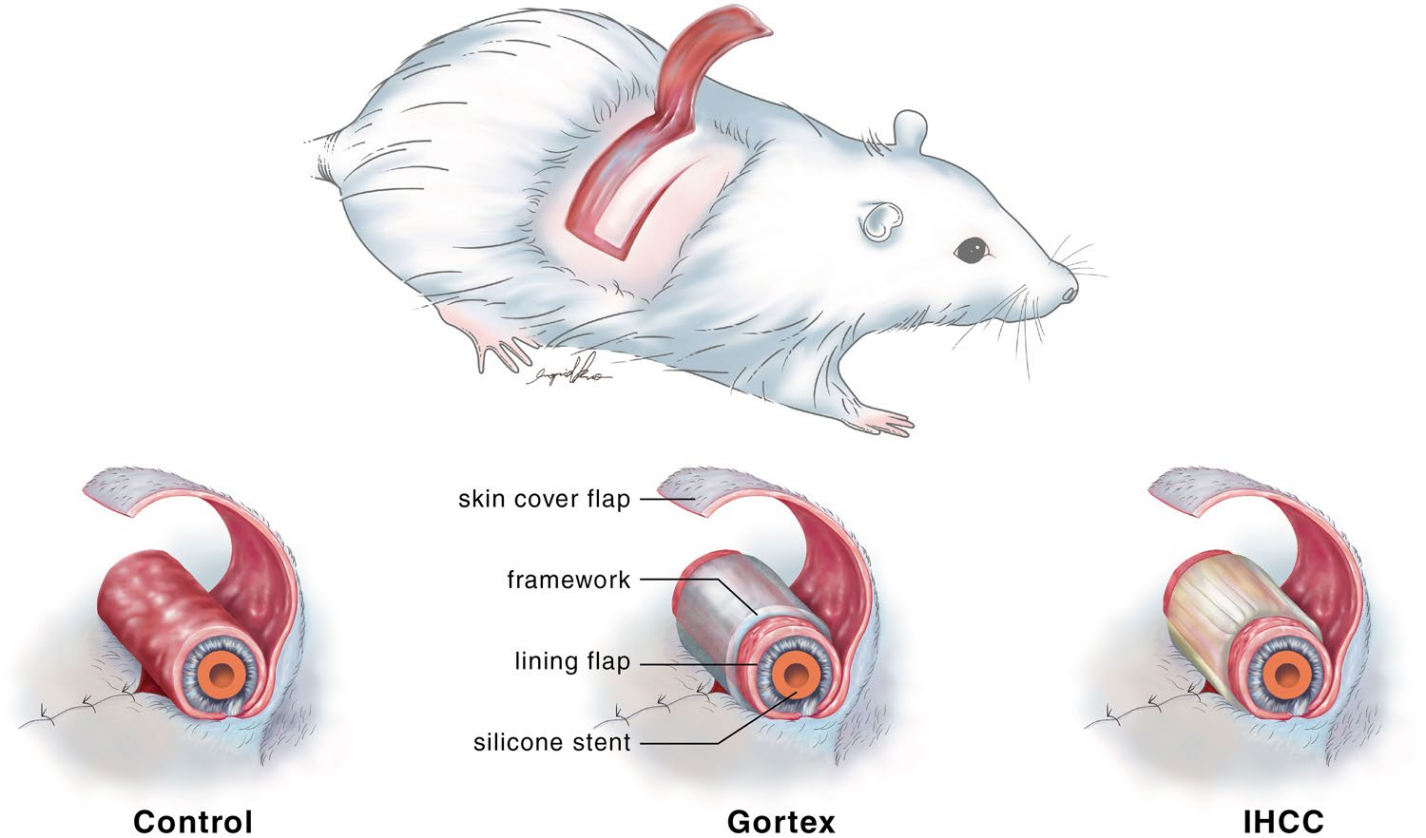
(Upper) A random fasciocutaneous flap was elevated. The flap was divided in half, to establish a lining and skin coverage. (Left, middle, right) In the control group, no framework was placed. In the experimental groups, ePTFE (Gortex) and IHCC were inserted between the lining and skin cover flaps as a framework. The tube structure was established to imitate the airway in nasal reconstruction.

**Figure 4:**
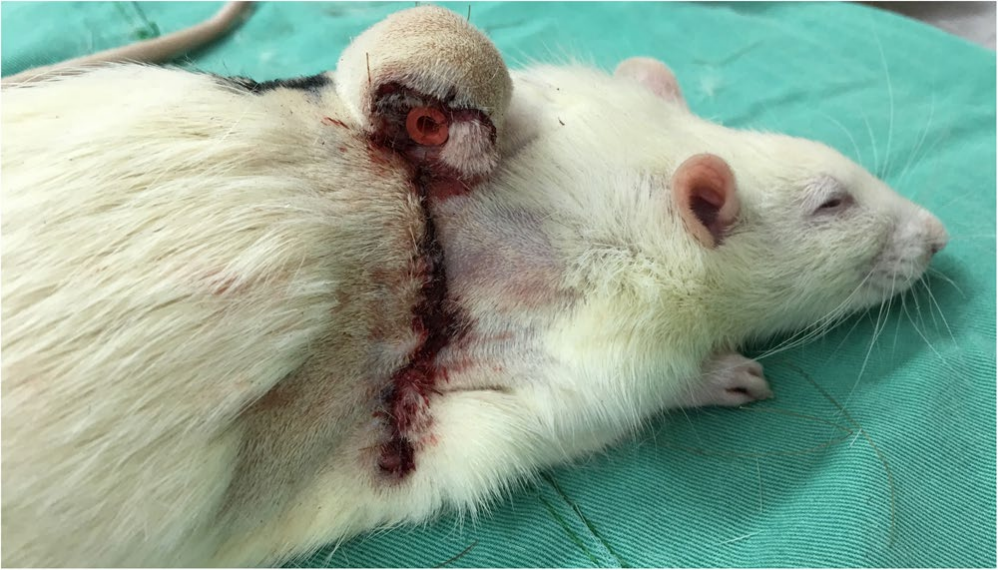
The construct was designed to imitate the airway in nasal reconstruction.

**Figure 5:**
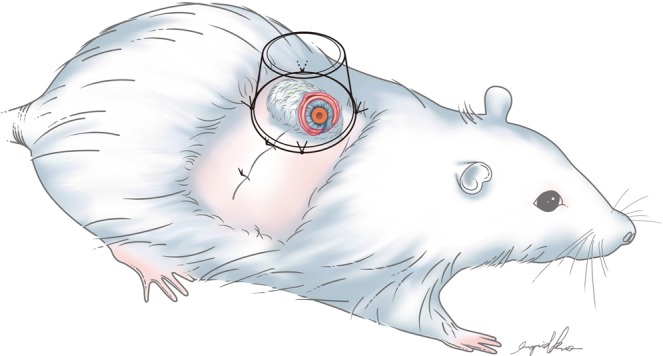
A plastic cup fixed with several stiches was placed as a shield to control for inevitable behavior such as hindquarter rubbing and rolling in contamination, and to prevent self-destruction of the tubed flap.

This study was performed at Chang Gung Memorial Hospital with approval from the Institutional Animal Care and Use Committee (approval number: 2014122209). All experiments were performed in accordance with relevant guidelines and regulations. Thirty Sprague-Dawley rats weighing 300–500 g were included initially. However, three rats died due to hypothermia and four rats died due to intolerance of multiple flaps harvested on the back simultaneously. Therefore, we improved the heat preservation system and modified the experiment design into harvesting one flap at one rat at a time. The remained twenty-three rats were randomly divided into three groups: a control group (n = 5) where no framework was placed, an IHCC group (Costal cartilage; LifeNet Health, Virginia Beach, VA) (n = 10), and ePTFE group (Gore-Tex, Implantech ePTFE sheeting, Implantech Associates, Inc., Ventura, CA) (n = 8). Every rat was anesthetized with isoflurane, dorsal hair was shaven and the skin was sterilized with povidone-iodine solution. A random-pattern fasciocutaneous flap measuring 4 cm x 3 cm was elevated. The donor site was closed primarily. The flap was divided longitudinally to create two flaps, each measuring 4 cm x 1.5 cm in size, one for the lining flap and the other skin cover **(**Fig. 3, upper). The lining flap was rolled and sutured to the flap base with the raw surface facing outwards. A 20-French silicone stent was placed to protect the simulated airway. **(**Fig. 3). For rats assigned to the control group, the skin cover flap was opposed to the lining flap without framework, and sutured with 5–0 chromic sutures **(**Fig. 3, left; Fig. 4**)**. In the other groups, ePTFE and IHCC rim grafts measuring 3 cm long x 1 cm wide x 2 mm thick were inserted between the inner lining flap and outer skin flap **(**Fig. 3, middle and right). The interface was closed to prevent graft exposure. After implantation, IV antibiotics (cefazolin sodium, 15 mg/kg) and analgesics (ketoprofen, 6.25 mg/kg) were injected subcutaneously. For three days, antibiotic was administered daily to prevent infection. To control for inevitable behavior such as hindquarter rubbing and rolling in contamination, and to prevent self-destruction of the tubed flap^14^, a plastic cup fixed with several stiches was placed as a shield **(**Fig. 5**)**.

### Evaluation of flap collapse

**Figure 6:**
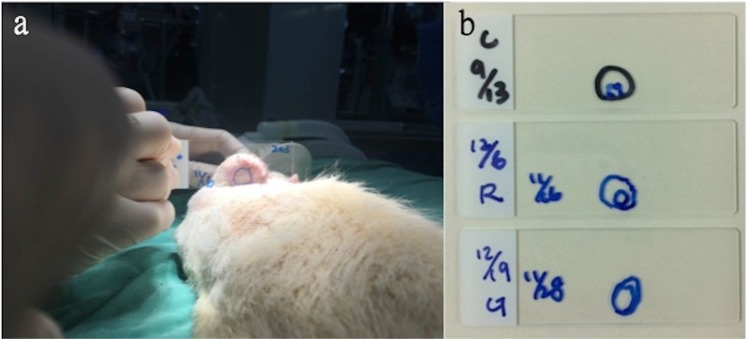
(**a**) The negative pressure suction system with continuous negative pressure was connected to one side of the tubing flap while the opposite side was sealed by a glass slide to create a vacuum. (**b**) The cross-sectional area of tube before and after simulated inhalation in three groups was documented. (upper: control group, middle: IHCC group, lower: ePTFE group).

Three weeks after flap elevation, rats were anesthetized and silicone tubes were removed. A surgical suction unit providing negative pressure suction system with continuous negative pressure of 60–70 mmHg was connected to one end of the tubed flap while the opposite side was sealed with a glass slide **(**Fig. 6a**)**. This model was designed to simulate the effect of negative pressure in the airway while breathing. We marked the outline of the tubed “airway” on the glass side before and after negative pressure was applied, and documented the change of the cross-sectional area of the tubed “airway” **(**Fig. 6b, Video 2). Cross-sectional area was measured using Image J software (version 1.50i 26 March 2016). The function of polygon selections was used for manually selecting the area of the cross-sectional area on the photos of glass slide. Then the analyze function was utilized for measuring the area which was chosen. Each measurement was performed three times and the average was used for analysis (Supplementary Fig. 1). The degree of airway collapse was calculated as a percentage of the original cross-sectional area before the application of negative pressure.

### Histological Evaluation

Rats were then sacrificed using a carbon dioxide chamber. Cross-sections of the composite flap were sectioned for hematoxylin and eosin (H&E) stain to evaluate histological change and tissue ingrowth.

### Statistical Analysis

Comparison of airway collapse between the groups was analyzed using the Mann-Whitney U test. Data were analyzed using SPSS for Windows, Version 20.0 (SPSS, Inc., Chicago, IL), and values of p < 0.05 were considered statistically significant.

**(**Informed consent has been obtained to publish all the identifying information/images (Video 1) in an online open-access publication.)

### Conclusions

This is the first animal model to simulate nasal airway reconstruction. ePTFE reinforcement is more effective in reducing cross sectional area when a vacuum is applied than irradiated cartilage.

## Supplementary information

video 1

video 2

supplementary file

## Data Availability

The datasets analyzed during the current study are available from the corresponding author on reasonable request.
