# Supplementary material for: Preventing nasal airway collapse with irradiated homologous costal cartilage versus expanded polytetrafluoroethylene: a novel animal model for nasal airway reconstruction
Source: Sci Rep. 2019 Apr 30;9:6670. doi: 10.1038/s41598-019-42947-8 (PMC6491583; doi:10.1038/s41598-019-42947-8)
Supplement: Supplementary file 3 — supplementary file [file 41598_2019_42947_MOESM3_ESM.pdf]

Title

Preventing nasal airway collapse with irradiated homologous costal cartilage versus extended polytetrafluoroethylene: a novel animal model for nasal airway reconstruction

Authors

Cheng-I Yen, M.D. [1]; Jonathan A. Zelken, M.D.[2], Chun-Shin Chang M.D. [1];

Hung-Chang Chen, M.D.[1]; Shih-Yi Yang, M.D. [1]; Shu-Yin Chang, M.D.[1];

Jui-Yung Yang, M.D. [1]; Shiow-Shuh Chuang, M.D. [1]; Yen-Chang Hsiao, M.D.[1*]

Affiliation

Department of Plastic and Reconstructive Surgery, Chang Gung Memorial Hospital, College of Medicine, Chang Gung University, Taipei, Taiwan[1]

Private Practice, Newport Beach, California, USA [2]

Corresponding Author

Yen-Chang, Hsiao, M.D.

Department of Plastic and Reconstructive Surgery, Chang Gung Memorial Hospital, Linkuo. (5, Fu-Hsin Street, Kwei-Shan, Taoyuan 333, Taiwan)

Tel: 886-3-328-1200, ext. 3221

E-mail: **b8301063@gmail.com**

Supplementary figure 1

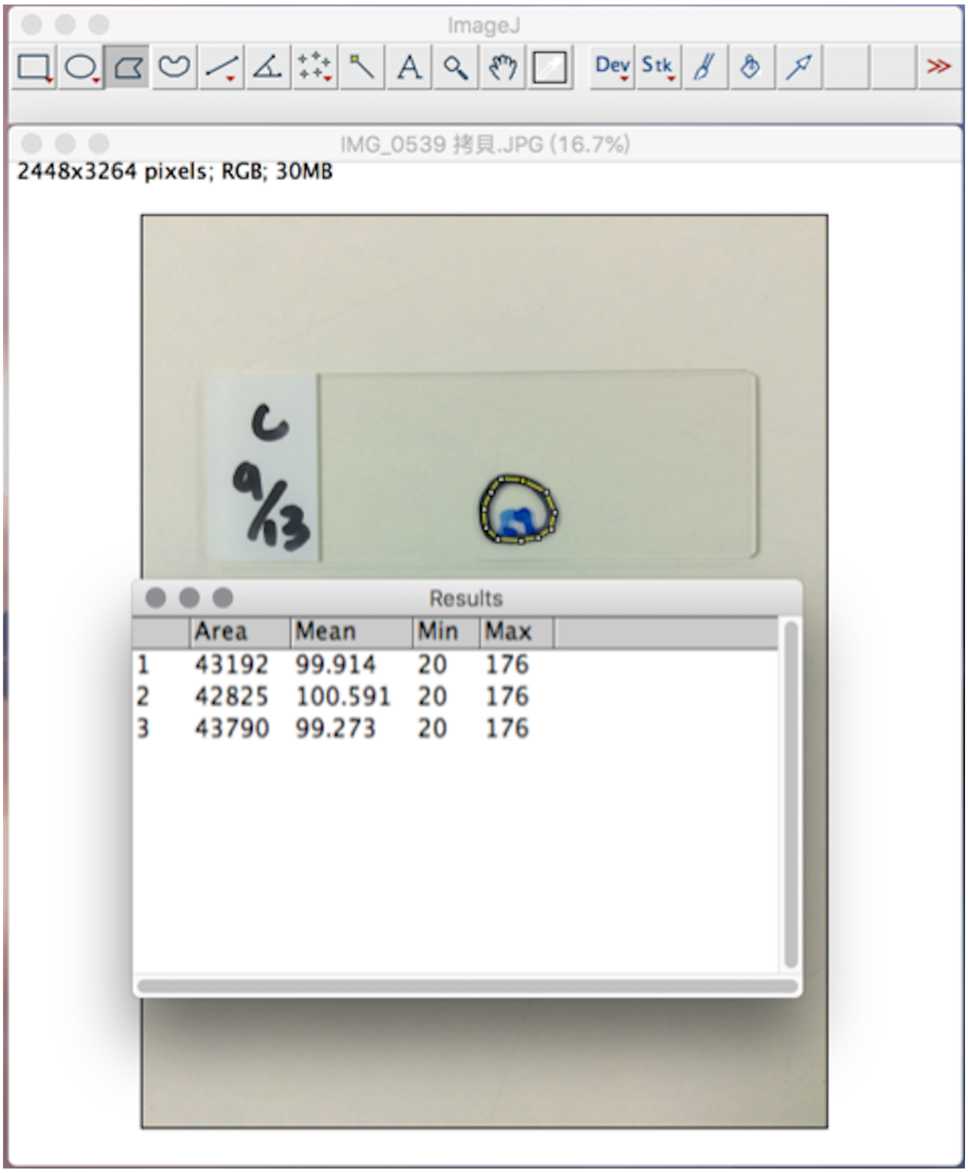

Supplementary figure 1. Cross-sectional area was measured using Image J software (version 1.50i 26 March 2016). The function of polygon selections was used for manually selecting the area of the cross-sectional area on the photos of glass slide. Then the analyze function was utilized for measuring the area which was chosen. Each measurement was performed three times and the average was used for analysis.

Video Legends

Video 1. A 67-year-old man with nasal defect after squamous cell carcinoma extirpation was reconstructed with forehead flap for outer skin and free flap for nasal lining. After operation, negative pressure in the airway while inhalation can overcome the support of the cartilage framework and tissue, and resulted in the collapse of airway.

Video 2. Different degree of flap collapse in the airway model between control, IHCC and ePTFE groups.
